# Outcomes and Diagnostic Processes in Outpatients with Presumptive Tuberculosis in Zomba District, Malawi

**DOI:** 10.1371/journal.pone.0141414

**Published:** 2015-11-10

**Authors:** Monique van Lettow, Richard Bedell, Sonia Maosa, Kenneth Phiri, Adrienne K. Chan, Edson Mwinjiwa, Aunex Kwekwesa, Harry Kawonga, Martias Joshua, Anthony D. Harries, Joep J. van Oosterhout

**Affiliations:** 1 Dignitas International, Zomba, Malawi; 2 Dalla Lana School of Public Health, University of Toronto, Toronto, Canada; 3 Division of Infectious Diseases, Sunnybrook Health Sciences Centre, University of Toronto, Toronto, Canada; 4 Zomba Central Hospital, Ministry of Health, Zomba, Malawi; 5 The International Union against Tuberculosis and Lung Disease, Paris, France; 6 London School of Hygiene and Tropical Medicine, London, United Kingdom; 7 Department of Medicine, College of Medicine, University of Malawi, Blantyre, Malawi; Universidad Nacional de la Plata, ARGENTINA

## Abstract

**Background:**

In Malawi, outpatients who have presumptive tuberculosis (TB), i.e. fever, night sweats, weight loss and/or any-duration cough (HIV-infected) or cough of at least 2 weeks (HIV-uninfected), are registered in chronic cough registers. They should receive a diagnostic work-up with first-step provider-initiated HIV testing and sputum testing which includes XpertMTB/RIF, following a national algorithm introduced in 2012.

**Methods:**

An operational study, in which we prospectively studied 6-month outcomes of adult outpatients who were registered in chronic cough registers in Zomba Central Hospital and Matawale peri-urban Health Center, between February and September 2013. We recorded implementation of the diagnostic protocol and outcomes at 6 months from registration.

**Results:**

Of 348 patients enrolled, 165(47%) were male, median age was 40 years, 72(21%) had previous TB. At registration 154(44%) were known HIV-positive, 34(10%) HIV-negative (26 unconfirmed) and 160(46%) had unknown HIV status; 104(56%) patients with unknown/unconfirmed HIV status underwent HIV testing. At 6 months 191(55%) were HIV-positive, 87(25%) HIV-negative (26 unconfirmed) and 70(20%) still had unknown HIV status. Higher age and registration in Matawale were independently associated with remaining unknown HIV status after 6 months. 62% of patients had sputum tested, including XpertMTB/RIF, according to the algorithm. TB was diagnosed in 54(15%) patients. This was based on XpertMTB/RIF results in 8(15%) diagnosed cases. In 26(48%) TB was diagnosed on clinical grounds. Coverage of ART in HIV-positive patients was 89%. At 6 months, 236(68%) were asymptomatic, 48(14%) symptomatic, 25(7%) had been lost-to-follow-up and 39(11%) had died. Mortality among those HIV-positive, HIV-negative and with unknown HIV-status was 15%, 2% and 10%, respectively. Male gender, being HIV-positive-not-on-ART and not receiving antibiotics were independent risk factors for mortality.

**Conclusion:**

HIV prevalence among patients with presumptive TB was high (55%). One quarter was not HIV tested and mortality in this group was substantial (10%). The impact of XpertMTB/RIF on TB diagnosis was limited.

## Introduction

The HIV/AIDS epidemic has increased the global tuberculosis (TB) burden greatly. Early detection of HIV infection among TB patients offers the opportunity to promptly link patients to HIV care interventions, such as cotrimoxazole prophylactic treatment (CPT) and antiretroviral treatment (ART), which can reduce morbidity and mortality [1]. Consequently, attention has focused on the necessity to coordinate TB and HIV/AIDS control programme services more closely [2].

Mortality among patients with presumptive TB, also known as TB suspects, is high. In Malawi, mortality rates among patients with presumptive TB range between 25–45% [3]. A study conducted at Zomba Central Hospital in 2010, reported that 29% of patients with presumptive tuberculosis died during the period they were admitted to hospital and being investigated for TB [4]. The same study also showed that of those who knew their HIV status (35%), all were HIV-positive, and that only few patients with unknown HIV status received provider initiated testing (PITC). High, undiagnosed, HIV-prevalence among patients with presumptive TB is thought to be responsible for the mortality in this group.

In high HIV and TB burden settings such as Malawi, policy makers have agreed that PITC should be promoted and provided not only to patients with active tuberculosis who register for treatment, but also to patients with presumptive TB [5,6]. The revised 2012 National Tuberculosis Program (NTP) manual recommends offering routine opt-out HIV testing to active and presumptive TB cases [7].

In Malawi, where the national adult HIV prevalence is 10% [8], TB cases are largely detected through passive case finding. Patients are considered to have presumptive tuberculosis if they report fever, night sweats, weight loss, and/or any-duration cough (HIV-infected persons) or cough of at least 2 weeks duration (HIV-uninfected persons). At health facilities, such patients are registered in standard chronic cough registers provided by the NTP. Patients with presumptive TB need to receive PITC and submit sputum specimens to health facility laboratories for smear microscopy for acid-fast bacilli (AFB). Patients whose sputum smears are negative for AFB should be managed according to an algorithm that includes empiric antibiotic treatment for bacterial causes of respiratory infection, and chest radiography in order to determine whether or not the patients have clinical/radiological evidence of smear-negative pulmonary TB.[7]

A rapid DNA-based method [XpertMTB/RIF] has demonstrated high sensitivity among HIV infected smear-negative pulmonary TB cases [9]. The authors found sensitivity for detection of smear-negative pulmonary TB of 72.5%, 85.1% and 90.2% with one, two and three tests per patient respectively. The use of XpertMTB/RIF would allow a more accurate diagnosis of smear-negative pulmonary TB, and have a beneficial effect on the diagnostic process for patients with presumed tuberculosis, of whom a large proportion have associated HIV-infection.

The revised 2012 NTP manual now recommends first-step provider-initiated HIV testing (PITC) and the use of Xpert MTB/RIF in a new diagnostic algorithm for patients with presumptive TB. To explore the application of this new algorithm in a real-life setting, we prospectively studied its implementation and the subsequent 6-month outcomes of patients registered in chronic cough registers in two clinics in Zomba District.

## Methods

### Study design and setting

We conducted an observational cohort study of adult outpatients (16 years or older) in Zomba Central Hospital (ZCH) and Matawale peri-urban Health Center (MHC), in the routine operational setting. We recruited participants at the point of registration in the chronic cough registers between February and September 2013. We recorded implementation of the diagnostic protocol and outcomes at 6 months from registration. The study sites, ZCH and MHC were purposively selected, because in both facilities an electronic patient unique identification system has been introduced. This facilitates tracking of referrals between the outpatient department and HIV and TB care services, but does not allow tracking referrals to other care services such as HIV testing and counselling (HTC) or laboratory services within the same facility. ZCH has XpertMTB/RIF facilities on-site, MHC sends samples to ZCH.

In 2012 a new diagnostic algorithm for presumptive TB patients was introduced in Malawi. The algorithm directs that: a) XpertMTB/RIF is indicated as the primary sputum diagnostic test for TB in patients with severe disease who need to be hospitalized, for retreatment cases and for those with otherwise suspected multidrug resistant tuberculosis; and b) for all others, XpertMTB/RIF should be reserved for when sputum is AFB negative [7]. If sputum microscopy and XpertMTB/RIF are negative, response to antibiotics and the result of a chest X-ray determine the diagnosis of smear negative pulmonary TB. National TB guidelines allow health facilities without Xpert MTB/RIF on site, such as MHC, to pursue treatment of other causes of the syndrome (mostly bacterial pneumonia and Pneumocystis pneumonia) before referring for XpertMTB/RIF testing. National guidelines were followed for rapid HIV antibody testing [10], and laboratory diagnostic sputum smear microscopy and Xpert MTB/RIF testing [7]. Microbiologically confirmed TB was defined as at least one positive sputum smear examination, or if *Mycobacterium TB* was identified from a sputum specimen by Xpert MTB/RIF.

### Data collection

After written informed consent, a structured piloted and pre-tested questionnaire was used at enrolment to ascertain socio-demographic characteristics, HIV status (if known), ART status (if known HIV-infected) and duration of cough. In addition, mobile phone numbers and residence location information were collected to enable follow up data collection at 6 months from registration.

Uptake and outcome variables were extracted from health facility registers (chronic cough register, TB laboratory register, laboratory register for XpertMTB/RIF test, X-ray, HTC, TB, ART) and where possible from the electronic data systems.

Primary outcomes of interest at 6-months from registration were *alive*, *dead*, and *lost to follow-up*. Secondary outcomes included the number of adults registered in the chronic cough register as diagnosed with active TB and the proportion that started anti-TB treatment, the number diagnosed with HIV and the proportion who were referred to HIV services and who started ART. The time from being registered in the cough register to the initiation of treatment of final diagnoses was also recorded.

Six months from time of registration/enrolment participants were contacted by phone and asked to return to the health facility at an arranged date to conduct a second face-to-face interview. In some cases, interviews were conducted by phone. If participants could not be reached by phone, they were traced and interviewed at their place of residence.

At the 6-month follow up visit, a structured piloted and pre-tested questionnaire was used to confirm and complete data on uptake and timing of HTC (referral and test results), diagnosis and uptake and timing of treatment for tuberculosis and HIV. Health passports were reviewed (when available) to verify information. In addition, the outcomes *alive without symptoms*, *alive and symptomatic*, *lost to follow up* and *death* were recorded.

### Data management and analysis

Data were double entered into a Microsoft Access database. Relevant data from the electronic monitoring systems at the sites were extracted and merged into the database. Descriptive analyses, using IBM SPSS Statistics 20 (IBM, Armonk, NY, USA) software, included medians with interquartile ranges [IQR], and proportions to summarize the data. To explore factors associated with having an unknown HIV-status, binary logistic regression models were fitted with having an unknown HIV-status at 6 months follow up (versus those having a confirmed result), as the outcome variable. To explore factors associated with mortality, binary logistic regression models were fitted with having died (versus being alive) within 6 months from registration in the chronic cough register as the outcome variable. Crude odds ratios (OR) and adjusted odds ratios (aORs) with 95% CIs were calculated. Adjustment for the following variables took place in both models: gender, age, site of registration in the chronic cough register, duration of cough and reported previous tuberculosis treatment. In addition, HIV (ART) status, sputum AFB results, XpertMTB/RIF results, having started and time to initiation of TB or antibiotic treatment were included in the model exploring associations with mortality. A significance level of 0.05 was set for all statistical testing.

### Ethical approval

Ethical approval was granted by the National Health Sciences Research Committee, Malawi (#1055) and the Ethics Advisory Group of the International Union Against Tuberculosis and Lung Disease, Paris, France (#106/12). In the Malawi health system, the age group 15–24 are defined as young adults, and were approved to be included in the study. However, only 2 participants were under the age of 18 years old, for whom written guardian witness was sought.

## Results

### Characteristics of study participants

A total of 348 adults were enrolled in the study; 165 (47%) were male, the median age was 40 (IQR 32–50). The median duration of cough was 3 weeks (IQR 2–4) and 72 (21%) had received prior TB treatment. Table 1 gives an overview of the characteristics of study participants and HIV status ascertainment by facility.

**Table 1:** Characteristics of study participants’ registered in chronic cough registers in Zomba Central Hospital and Matawale Health Centre between February and September 2013.

Characteristics by facility *at registration*	ZCH*n = 257	MHC*n = 91	Total n = 348
Male gender (n) %	(114) 44%	(51) 56%	(165) 47%
Age (median; IQR)	39 (32–50)	40 (32–50)	40 (32–50)
Duration of cough in weeks (median; IQR)	3 (2–4)	3 (2–6)	3 (2–4)
Previous TB (n) %	(57) 22%	(15) 16%	(72) 21%
**HIV status *at registration***			
HIV-infected (n) %	(123) 48%	(31) 34%	(154) 44%
HIV-uninfected, confirmed (n) %	(6) 2%	(2) 2%	(8) 2%
HIV-uninfected, reported (n) %	(26) 10%	-	(26) 8%
HIV status unknown (n) %	(102) 40%	(58) 64%	(160) 46%
**HIV status *at 6 months follow-up***			
HIV-infected (n) %	(152) 59%	(39) 43%	(191) 55%
HIV-uninfected, confirmed (n) %	(38) 15%	(23) 25%	(61) 17%
HIV-uninfected, reported (n) %	(26) 10%	-	(26) 8%
HIV status unknown (n) %	(41) 16%	(29) 32%	(70) 20%

* ZCH: Zomba Central Hospital; MCH: Matawale Health Centre

### HIV status ascertained from registration in chronic cough register through 6-month follow up

At registration in both study sites 154 (44%) adults were known HIV positive, 34 (10%) were HIV negative (8/34 confirmed) and 160 (46%) were HIV unknown. Between registration and the 6-month follow up point, 104 (56% of unknown and unconfirmed) underwent HIV testing. At 6 months, a total of 191 (55%) adults were confirmed HIV positive, 61 (18%) confirmed negative, 26 (7%) unconfirmed negative and 70 (20%) remained HIV unknown. Higher age and registration in MHC were independently associated with remaining HIV unknown after 6 months (Table 2).

**Table 2:** Factors associated with having an unknown HIV-status at 6 month follow up.

	Univariate O.R. (95% C.I.)	P-value	Multivariate O.R. (95% C.I.)*	P-value
Gender (Male)	1.41 (0.83–2.39)	0.20		
Age (per year)	1.03 (1.01–1.05)	0.001	1.04 (1.02–1.06)	0.001
Site**:				
ZCH	-	-	-	-
MHC	2.46 (1.42–4.28)	0.001	2.56 (1.42–4.62)	0.002
Duration of cough (in weeks)	1.00 (0.97–1.04)	0.88		
Previous TB (yes)	0.69 (0.34–1.39)	0.30		

* Adjusted for all other variables in the binary logistic regression model. In the multivariate analysis, only variables with significant associations are shown.

** ZCH: Zomba Central Hospital; MCH: Matawale Health Centre

### Tuberculosis diagnostic pathways

**Fig 1:**
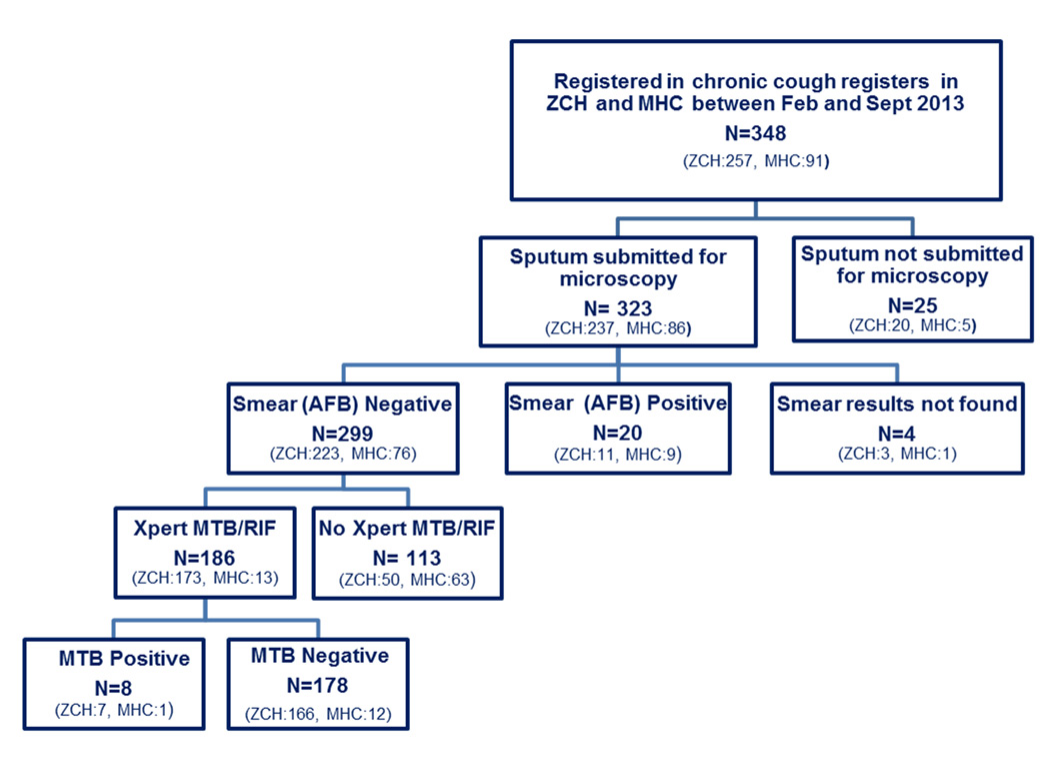
Laboratory tests conducted to identify pulmonary TB in adults registered in chronic cough registers in Zomba Central Hospital and Matawale Health Centre.

Fig 1 shows the laboratory evaluation conducted to identify pulmonary TB among the study participants in both sites. Of 348 adults registered in the chronic cough registers, 323 reported submission of sputum for microscopy. Of these, 319 AFB results were found in the TB laboratory registers (20 AFB+/299 AFB-). 12/20 (60%) of adults with a positive smear result were HIV positive. Out of the 299 AFB negative smear results, 186 (62%) had XpertMTB/RIF following sputum smear examination according to the protocol (72% in ZCH; 24% in MHC). Tuberculosis diagnosis was made in 54/348 (15%). In 8/54 (15%) cases the diagnosis was based on the Xpert MTB/RIF result and in 26/54 (48%) TB treatment was started on the basis of clinical diagnosis. 28/54 (52%) Patients had microbiologically confirmed TB diagnosis (20 AFB+ and 8 XpertMTB/RIF+); there were no cases of rifampicin resistance during Xpert MTB/RIF testing.

### 6-month outcomes of patients with presumptive tuberculosis in ZCH and MHC by diagnostic process and treatment uptake

Table 3 shows 6-months outcomes by site and HIV and ART status. At 6 months, 236 (68%) of all patients were asymptomatic, 48 (14%) were symptomatic, 25 (7%) had been lost-to-follow-up and 39 (11%) of all patients had died (12% in ZCH; 8% in MHC). Mortality among those HIV-positive, HIV-negative and with unknown HIV-status was 15%, 2% and 10% respectively.

**Table 3:** Six-month outcomes of patients with presumptive tuberculosis in Zomba Central Hospital and Matawale Health Centre by HIV/ART status.

Outcomes (n) %	HIV+ on ART		HIV+ not on ART		HIV		HIV status Unknown		TOTAL	
Alive asymptomatic	(121)	72%	(13)	62%	(49)	80%	(53)	55%	**(236)**	**68%**
Alive symptomatic	(18)	11%	(1)	5%	(11)	18%	(18)	19%	**(48)**	**14%**
Lost-to-follow-up	(8)	5%	(1)	5%			(16)	16%	**(25)**	**7%**
Died	(22)	13%	(6)	29%	(1)	2%	(10)	10%	**(39)**	**11%**
**Total**	**169**		**21**		**61**		**97**		**348**	

Table 4 provides details of the diagnostic processes, treatment uptake and time to treatment of final diagnoses by HIV (ART) status, and subsequent 6-month outcomes. Coverage of ART in confirmed HIV positive patients was 89%. TB treatment was started in 53/54 (98%) adults diagnosed with TB and among those, 32/53 (60%) also received antibiotic treatment. There were 219 presumed TB patients who received treatment for bacterial infection only, and for 76, no treatment was reported. In multivariate analysis, male gender, being HIV positive but not on ART and not having started antibiotics were independent risk factors for mortality (Table 5).

**Table 4:** Six-month outcomes of patients with presumptive tuberculosis in Zomba Central Hospital and Matawale Health Centre by diagnostic process and treatment uptake.

Diagnostic process	Treatment Uptake	n = 348	Time to treatment, median days (IQR)	HIV (ART) status	LTF	Alive	Died
**Sputum not submitted for microscopy or not found,**	Treatment for Bacterial Infection only	17	0 (0–19)	HIV+ on ART		8	
** N = 29**				HIV+ not on ART		3	
** **				HIV -		1	
** **				Unknown		5	
** **	No treatment reported	12	-	HIV+ on ART	1	1	3 (60%)
** **				HIV+ not on ART		1	
** **				Unknown	3	1	2 (33%)
**Smear microscopy (AFB) positive,**	TB treatment on basis of AFB results	19	4 (1–11)	HIV+ on ART		10	1 (9%)
**N = 20**				HIV -		6	
** **				Unknown		2	
** **	No treatment reported	1	-	HIV+ not on ART			1(100%)
**Smear microscopy (AFB) negative; Xpert MTB+,**	TB treatment on basis of GeneXpert results	8	3 (2–19)	HIV+ on ART		4	
**N = 8**				HIV+ not on ART			1(100%)
** **				HIV -		3	
**Smear microscopy (AFB) negative; Xpert MTB-,**	TB treatment on clinical grounds	13	6 (3–8)	HIV+ on ART		6	1 (14%)
** N = 165**				HIV+ not on ART		1	1 (50%)
** **				HIV -		2	
** **				Unknown		2	
** **	Treatment for Bacterial Infection only	114	2 (0–21)	HIV+ on ART		56	3 (5%)
** **				HIV+ not on ART		4	1 (20%)
** **				HIV -		21	
** **				Unknown		27	2 (7%)
** **	No treatment reported	38	-	HIV+ on ART	5	8	7 (35%)
** **				HIV+ not on ART	1		1 (50%)
** **				HIV -		2	
** **				Unknown	4	7	3 (21%)
**Smear microscopy (AFB) negative—Xpert result not found,**	TB treatment on clinical grounds	1	24	HIV+ on ART		1	
**N = 13 **	Treatment for Bacterial Infection only	9	0 (0–3)	HIV+ on ART		1	3 (75%)
** **				HIV -		3	
** **				unknown		1	1 (50%)
** **	No treatment reported	3	-	HIV+ on ART	1		
** **				unknown	2		
**Smear microscopy (AFB) negative—. No Xpert,**	TB treatment on clinical grounds	12	6 (4–8)	HIV+ on ART		7	2 (22%)
** N = 113**				HIV -		1	1 (50%)
** **				unknown		1	
** **	Treatment for Bacterial Infection only	79	3 (0–29)	HIV+ on ART		34	2 (6%)
** **				HIV+ not on ART		5	
** **				HIV -		18	
** **				unknown		20	
** **	No treatment reported	22	-	HIV+ on ART	1	3	
** **				HIV+ not on ART			1(100%)
** **				HIV -		3	
** **				unknown	7	5	2 (14%)

**Table 5:** Factors associated with Mortality.

	Univariate O.R. (95% C.I.)	P-value	Multivariate O.R. (95% C.I.)*	P-value
Gender (male)	2.78 (1.35–5.70)	0.005	2.98 (1.31–6.77)	0.009
Age (per year)	1.01 (0.99–1.04)	0.25		
**Site** **:				
ZCH	-			
MHC	0.56 (0.24–1.33)	0.19		
**HIV (ART) status** at 6 months:				
HIV+ on ART	9.43 (1.24–71.5)	0.03	8.33 (1.01–67.2)	0.05
HIV+ not on ART	25.7 (2.8–99.9)	0.004	33.5 (3.26–99)	0.003
HIV-	-	-	-	-
unknown	8.33 (1.04–67.0)	0.04	5.3 (0.6–45)	0.13
Duration of cough (in weeks)	1.00 (0.96–1.04)	0.94		
Previous TB (yes)	0.53 (0.20–1.40)	0.20		
**Sputum AFB results**				
AFB+	0.42 (0.07–2.46)	0.34		
AFB-	0.49 (0.17–1.40)	0.18		
no result	-	-		
**GeneXpert results**				
MTB+	1.84 (0.37–9.25)	0.46		
MTB-	1.22 (0.61–2.43)	0.57		
no result	-	-		
Having started TB Tx	1.14 (.048–2.74)	0.77		
Having started AB Tx	0.10 (0.05–0.22)	0.001	0.11 (0.05–0.24)	0.001
Time to treatment (TB or AB) (in days)***	0.99 (0.98–1.02)	0.91		

* Adjusted for all other variables in the binary logistic regression model. In the multivariable analysis, variables with significant associations are shown only.

** ZCH: Zomba Central Hospital; MCH: Matawale Health Centre

*** for final diagnoses; in case that TB and AB treatment were started, time to TB treatment was used

## Discussion

We found that the HIV prevalence in Malawian adult TB suspects was 55%. This falls within a range of studies on TB suspects from Kenya (n = 5457, HIV prevalence 62%, HIV unknown 11%), Uganda (n = 665, HIV prevalence 42%, HIV unknown 15% [11]), and Ethiopia (n = 506, HIV prevalence 27%, HIV unknown 41% [12]). Despite differences in the design of the studies and the study settings, our study confirms that HIV prevalence among presumptive TB patients in sub-Saharan Africa is very high.

Because the high mortality among patients with presumed TB is believed to be associated with undiagnosed HIV infection, the Malawi NTP changed their guidelines in 2012, placing PITC as a prominent first step in the diagnostic algorithm. Probably due to this measure, HIV testing uptake and the prevalence of known HIV status were improved (80% vs. 44%) compared to our study in ZCH from 2011 [4]. This reflects growing experience with the introduction of PITC in various circumstances in resource limited settings, as summarized in a systematic review [13]. However, we believe that the rate of unknown HIV status six months after registration remained unacceptably high and there was substantial mortality in this group (10%). It is likely that HIV prevalence in those with unknown HIV status was considerable and that most would have been eligible for CPT and ART, both of which reduce mortality. In a survey in northern Malawi, more than 50% of HIV positive TB suspects were eligible for ART in 2006, when the CD4 threshold value for ART eligibility was lower than in the study period (CD4 <200 vs. <350 cells/μL) [14]. More effort is required to improve the uptake of HIV testing among TB suspects to allow initiation of ART and CPT, possibly even before the diagnostic work-up for TB has been completed [15]. In multivariate analysis, older age and being registered at MHC were associated with having unknown HIV status in our study. Factors such as free of charge availability of both HIV testing and treatment (as present in our setting) have earlier been identified as enablers to HIV testing [16]. We did not study the reasons why individual participants were not tested and we were unable to determine important barriers to HIV testing e.g. stigma, lack of a supportive social network (which may be more common in males) and health system related issues such as stock-outs of HIV test kits. Importantly, presumptive TB patients may not perceive themselves as being at high risk of HIV infection, which reduces willingness to undergo testing [16]. The high HIV prevalence in our local setting could be communicated to this specific group of patients to increase the chance of HIV testing uptake.

Measures to improve uptake of HIV treatment among TB patients have been successful in Malawi. Similar to the experience from South Africa [17], priority for PITC among patients who have been newly diagnosed with tuberculosis has led to high rates of ascertainment of HIV status (93% at national level), successful linkage to ART services (95% at national level) [18] and consequently to improvement in treatment outcomes [19, 20]. However, the cascade of HIV-TB interventions that leads to better outcomes should start before the diagnosis of TB is made and our results show that mortality during the diagnostic process is still considerable, even among outpatients. We found that male gender and being HIV positive-not on ART were independent risk factors for mortality. Men present later for HIV testing and care and tuberculosis diagnosis and treatment than women, and–probably as a consequence—have higher mortality rates during treatment for both conditions. Qualitative research in Malawi indicated that the perceived paramount importance of men’s role as breadwinner and the associated high opportunity costs of acknowledging illness may explain delays in seeking care for HIV and TB [21]. Although being HIV positive-on ART as well as having an HIV unknown status were also associated with mortality in univariate analyses, only a borderline association of being HIV positive and on ART remained in the multivariate model.

Not receiving antibiotic treatment was associated with mortality among TB suspects. This suggests that concomitant severe infections other than TB were important causes of death. A study conducted in Malawi in 2010 found that bloodstream infections, most involving non-typhoidal salmonellae, were common among HIV-infected, smear negative patients with fever and/or weight loss [22]. Receiving antibiotics may also be indirect evidence of more contact with health care providers leading to more timely treatment interventions. There is no evidence that the use of antibiotic treatment can lead to false negative results in gene Xpert MTB/RIF. We did not consistently collect information on the type of antibiotics used and our data did not allow us to distinguish the impact of antibiotic usage between those with and without a TB diagnosis. Further studies are required to determine if a routine course of broad-spectrum antibiotics is beneficial for patients undergoing investigation in chronic cough registers

In accordance with several other studies in operational settings, we did not see an association between XpertMTB/RIF test results and mortality reduction. A multi-center pragmatic trial from South Africa, Zambia, Zimbabwe and Tanzania found that in comparison with microscopy-based diagnosis, XpertMTB/RIF testing in primary health care settings allowed TB treatment to be started more frequently and earlier, but this had no effect on mortality [23]. In a randomized trial from South Africa, Xpert MTB/RIF testing was also compared with a routine diagnostic algorithm in a primary health care setting. Xpert MTB/RIF testing led to increased microbiologically confirmed TB cases, more TB treatment initiations and an earlier start of TB treatment, but again without any effect on 6-month mortality [24]. In a Ugandan before/after study, the introduction of XpertMTB/RIF in a hospital setting reduced time to TB diagnosis, but not time to tuberculosis treatment initiation, and there was no effect on 2-month mortality. Similar to our study, this last study lacked statistical power to study impact on survival [25]. These studies point to the diagnostic benefits of XpertMTB/RIF, especially in HIV-infected TB suspects, but leave doubt about clinical benefits. This is probably due to the fact that clinicians in sub-Saharan Africa are prepared to start empirical TB treatment frequently in AFB smear negative TB suspects, which may also be the case in Malawi. More work is therefore required to evaluate the impact of XpertMTB/RIF testing in Malawi, especially in terms of patient outcomes. We did not find any cases of rifampicin resistance, which is consistent with a recent survey that showed that drug resistant TB is as low as 0.4% in new cases in Malawi [26].

The diagnostic impact of XpertMTB/RIF testing appeared modest in our real-life setting. This was mainly determined by the low uptake as only 186/328 (57%) of those eligible in both health facilities underwent Xpert MTB/RIF testing. Although samples from patients at MHC could be sent for Xpert MTB/RIF testing at the nearby ZCH, only 17% of samples from AFB sputum smear negative patients were sent. Apart from the logistical difficulties of sending samples to another health facility in the case of MHC, other factors may have played a role. Despite extensive off-site training, health care workers may not have fully understood or applied the algorithm. The volume of left-over sputum may have been insufficient for Xpert MTB/RIF testing after sputum was used for smear microscopy. Equipment may have been out of order and cartridges out of stock. A limitation of our study is that it did not address the causes of the low uptake of XpertMTB/RIF testing. Simplification of the algorithm by using XpertMTB/RIF testing as the primary test for all TB suspects, replacing smear microscopy, could facilitate uptake. However, in a low-income country like Malawi this option is unlikely affordable.

The prevalence of a positive XpertMTB/RIF test result was low (8/186; 4%) and the contribution of a positive XpertMTB/RIF result to TB diagnosis was moderate: 8/54 (15%). We could not determine the importance clinicians attached to a negative XpertMTB/RIF result in the diagnostic work-up. However, the low rate of clinical TB diagnoses among XpertMTB/RIF negative tuberculosis suspects (13/165; 8%) in combination with the low mortality in this group (2/165; 1%) suggests that a negative result gave clinicians confidence to diagnose and treat other conditions successfully.

We believe that our results may be representative for urban and peri-urban sites in Malawi, where many TB suspects are managed. Given that the TB and HIV services of the Ministry of Health in the study setting are supported by an NGO with a long-term presence in the area, we do not expect that results elsewhere in Malawi would be much better.

## Conclusion

We found high HIV prevalence in adult Malawian outpatients with presumptive TB. However, one quarter of patients was not HIV tested and in this group mortality was substantial. The uptake and impact of XpertMTB/RIF testing on TB diagnosis was limited and did not appear to influence survival. The new national TB diagnostic algorithm needs reinforcement in these clinics and its effectiveness requires evaluation on a wider scale.

## Supporting Information

S1 Dataset Dataset. (XLS)

## References

[1] Havlir DV, Getahun H, Sanne I, Nunn P. Opportunities and challenges for HIV care in overlapping HIV and TB epidemics. JAMA 2008; 300: 423–430. 10.1001/jama.300.4.423 18647985

[2] Corbett EL, Watt CJ, Walker N, Maher D, Williams BG, Raviglione MC, et al The growing burden of tuberculosis: global trends and interactions with the HIV epidemic. Arch Intern Med 2003; 63(9):1009–21. 10.1001/archinte.163.9.1009 12742798

[3] Hargreaves NJ, Harries AD, Kemp JR, Kwanjana JH, Salaniponi FM. Smear negative pulmonary tuberculosis: defining better approaches to case finding in Malawi. Malawi Med J 2002; 13: 20–22.

[4] Gawa LG, Reid T, Edginton ME, Van Lettow M, Joshua M, Harries AD. Diagnostic management and outcomes of pulmonary tuberculosis suspects admitted to a central hospital in Malawi. Public Health Action 2011; 1:2–5. 10.5588/pha.11.0007 26392925 PMC4547185

[5] Harries AD. Paying attention to tuberculosis suspects whose sputum smears are negative. J Tuberc Lung Dis 2011; 15(4):427–8. 10.5588/ijtld.11.0051 21396196

[6] World Health Organization. WHO policy on collaborative TB/HIV activities. Guidelines for national programmes and other stakeholders. Geneva, Switzerland, 2012. Available: http://whqlibdoc.who.int/publications/2012/9789241503006_eng.pdf. 23586124

[7] Malawi Ministry of Health. National TB Control Program Operational Manual. Ministry of Health, Malawi, 7th edition 2012 Available: http://www.medcol.mw/globalhealth/uploads/MalawianTBguidelines2012.pdf.

[8] UNAIDS. HIV and AIDS estimates (2014). Available: http://www.unaids.org/en/regionscountries/countries/malawi.

[9] Boehme CC, Nabeta P, Hillemann D, Nicol MP, Shenai S, Krapp F, et al Rapid molecular detection of tuberculosis and rifampin resistance. N Engl J Med 2010; 363:1005–101510. 10.1056/NEJMoa0907847 20825313 PMC2947799

[10] Malawi Ministry of Health. Clinical management of HIV in children and adults. 2014. Lilongwe, Malawi. Available: http://www.emtct-iatt.org/wp-content/uploads/2015/09/Malawi-HIV-Guidelines-2014.pdf.

[11] Odhiambo J, Kizito W, Njoroge A, Wambua N, Nganga L, Mburu M, et al Provider-initiated HIV testing and counselling for TB patients and suspects in Nairobi, Kenya. Int J Tuberc Lung Dis 2008; 12(3 Suppl 1):63–8. 18302825

[12] Deribew A, Negussu N, Melaku Z, Deribe K. Investigation outcomes of tuberculosis suspects in the health centers of Addis Ababa, Ethiopia. PLoS One 2011; 6(4):e18614 10.1371/journal.pone.0018614 21526179 PMC3079716

[13] Kennedy CE, Fonner VA, Sweat MD, Okero FA, Baggaley R, O'Reilly KR. Provider-initiated HIV testing and counseling in low- and middle-income countries: a systematic review. AIDS Behav 2013; 17(5):1571–90. 10.1007/s10461-012-0241-y 22752501 PMC3927322

[14] Munthali L, Mwaungulu JN, Munthali K, Bowie C, Crampin AC. Using tuberculosis suspects to identify patients eligible for antiretroviral treatment. Int J Tuberc Lung Dis 2006;10(2):199–202. 16499261

[15] Kerkhoff AD, Wood R, Lawn SD. Optimum time to start antiretroviral therapy in patients with HIV-associated tuberculosis: before or after tuberculosis diagnosis? AIDS 2011 10.1097/QAD.0b013e328345ee32 21346513

[16] Musheke M, Ntalasha H, Gari S, McKenzie O, Bond V, Martin-Hilber A, et al A systematic review of qualitative findings on factors enabling and deterring uptake of HIV testing in Sub-Saharan Africa. BMC Public Health 2013 13:220 10.1186/1471-2458-13-220 23497196 PMC3610106

[17] Lawn SD, Fraenzel A, Kranzer K, Caldwell J, Bekker LG, Wood R. Provider-initiated HIV testing increases access of patients with HIV-associated tuberculosis to antiretroviral treatment. S Afr Med J 2011; 101(4):258–62. 21786731 10.7196/samj.4392

[18] Government of Malawi, Ministry of Health. Integrated HIV-Programme report July-September 2014. Lilongwe, January 2015.

[19] Houben RM, Glynn JR, Mboma S, Mzemba T, Mwaungulu NJ, Mwaungulu L, et al The impact of HIV and ART on recurrent tuberculosis in a sub-Saharan setting. AIDS 2012; 26(17):2233–9. 10.1097/QAD.0b013e32835958ed 22951633

[20] Abdool Karim SS, Naidoo K, Grobler A, Padayatchi N, Baxter C, Gray A, et al Timing of initiation of antiretroviral drugs during tuberculosis therapy. N Engl J Med 2010; 362(8):697–706. 10.1056/NEJMoa0905848 20181971 PMC3076221

[21] Chikovore J, Hart G, Kumwenda M, Chipungu GA, Corbett L. ‘For a mere cough, men must just chew Conjex, gain strength, and continue working’: the provider construction and tuberculosis care-seeking implications in Blantyre, Malawi. Glob Health Action 2015, 8: 26292— 10.3402/gha.v8.26292 10.3402/gha.v8.26292 25833138 PMC4382597

[22] van Lettow M, Akesson A, Martiniuk ALC, Ramsay A, Chan AK, Anderson ST, et al Six-Month Mortality among HIV-Infected Adults Presenting for Antiretroviral Therapy with Unexplained Weight Loss, Chronic Fever or Chronic Diarrhea in Malawi. PLoS ONE 2012; 7(11): e48856 10.1371/journal.pone.0048856 23185278 PMC3501502

[23] Theron G, Zijenah L, Chanda D, Clowes P, Rachow A, Lesosky M, et al Feasibility, accuracy, and clinical effect of point-of-care Xpert MTB/RIF testing for tuberculosis in primary-care settings in Africa: a multicentre, randomised, controlled trial. Lancet 2014; 383(9915):424–35. 10.1016/S0140-6736(13)62073-5 24176144

[24] Cox HS, Mbhele S, Mohess N, Whitelaw A, Muller O, Zemanay W, et al Impact of Xpert MTB/RIF for TB diagnosis in a primary care clinic with high TB and HIV prevalence in South Africa: a pragmatic randomised trial. PLoS Med 2014; 11(11):e1001760 10.1371/journal.pmed.1001760 eCollection 2014. 25423041 PMC4244039

[25] Yoon C, Cattamanchi A, Davis JL, Worodria W, den Boon S, Kalema N, et al Impact of Xpert MTB/RIF Testing on Tuberculosis Management and Outcomes in Hospitalized Patients in Uganda. PLoS ONE 2012; 7(11): e48599 10.1371/journal.pone.0048599 23139799 PMC3490868

[26] Abouyannis M, Dacombe R, Dambe I, Mpunga J, Faragher B, Gausi F, et al Drug resistance of Mycobacterium tuberculosis in Malawi: a cross-sectional survey. Bull World Health Organ 2014 11 1;92(11):798–806. 10.2471/BLT.13.126532 25378741 PMC4221759

